# Biphasic glucose-stimulated insulin secretion over decades: a journey from measurements and modeling to mechanistic insights

**DOI:** 10.1093/lifemeta/loae038

**Published:** 2024-11-19

**Authors:** Xiaohong Peng, Kai Wang, Liangyi Chen

**Affiliations:** New Cornerstone Science Laboratory, State Key Laboratory of Membrane Biology, Beijing Key Laboratory of Cardiometabolic Molecular Medicine, Institute of Molecular Medicine, National Biomedical Imaging Center, The Beijing Laboratory of Biomedical Imaging, Peking-Tsinghua Center for Life Sciences, School of Future Technology, Peking University, Beijing 100871, China; Department of Physiology and Pathophysiology, School of Basic Medical Sciences, Peking University, Beijing 100191, China; Department of Physiology and Pathophysiology, School of Basic Medical Sciences, Peking University, Beijing 100191, China; New Cornerstone Science Laboratory, State Key Laboratory of Membrane Biology, Beijing Key Laboratory of Cardiometabolic Molecular Medicine, Institute of Molecular Medicine, National Biomedical Imaging Center, The Beijing Laboratory of Biomedical Imaging, Peking-Tsinghua Center for Life Sciences, School of Future Technology, Peking University, Beijing 100871, China; PKU-IDG/McGovern Institute for Brain Research, Beijing 100871, China

**Keywords:** glucose-stimulated insulin secretion, vesicle pools, readily releasable β-cells, β-cell heterogeneity

## Abstract

Glucose-stimulated insulin release from pancreatic β-cells is critical for maintaining blood glucose homeostasis. An abrupt increase in blood glucose concentration evokes a rapid and transient rise in insulin secretion followed by a prolonged, slower phase. A diminished first phase is one of the earliest indicators of β-cell dysfunction in individuals predisposed to develop type 2 diabetes. Consequently, researchers have explored the underlying mechanisms for decades, starting with plasma insulin measurements under physiological conditions and advancing to single-vesicle exocytosis measurements in individual β-cells combined with molecular manipulations. Based on a chain of evidence gathered from genetic manipulation to *in vivo* mouse phenotyping, a widely accepted theory posits that distinct functional insulin vesicle pools in β-cells regulate biphasic glucose-stimulated insulin secretion (GSIS) via activation of different metabolic signal pathways. Recently, we developed a high-resolution imaging technique to visualize single vesicle exocytosis from β-cells within an intact islet. Our findings reveal that β-cells within the islet exhibit heterogeneity in their secretory capabilities, which also differs from the heterogeneous Ca^2+^ signals observed in islet β-cells in response to glucose stimulation. Most importantly, we demonstrate that biphasic GSIS emerges from the interactions among α-, β-, and δ-cells within the islet and is driven by a small subset of hypersecretory β-cells. Finally, we propose that a shift from reductionism to holism may be required to fully understand the etiology of complex diseases such as diabetes.

## Introduction

Type 2 diabetes (T2D), characterized by hyperglycemia, poses a significant global health concern, currently affecting approximately 537 million individuals worldwide [1]. The pancreatic islets, mainly comprising α-, β-, δ-, PP, and ghrelin cells, play a crucial role in regulating glucose levels by secreting glucagon, insulin, somatostatin (SST), pancreatic polypeptide, and ghrelin, respectively [2]. Following food indigestion and subsequent plasma glucose elevation, insulin is secreted from pancreatic β-cells into the bloodstream, facilitating glucose uptake and utilization in downstream organs such as the liver, muscle, and adipocytes [3]. Originating primarily from obesity and multi-organ insulin resistance (IR), abnormalities in insulin secretion and β-cell function mark the critical transition from prediabetes to T2D [4]. Thus, deciphering the mechanisms regulating β-cell secretory function is essential for improving the treatment and prevention of the disease.

To meet various demands under different circumstances, insulin secretion from pancreatic β-cells is a tightly regulated process. At the single-cell level, β-cells are regulated by nutrient stimuli including glucose, fatty acids, and amino acids [5, 6]. Within the islet, their functions are also modulated by autocrine and paracrine interactions, such as glucagon, SST, and urocortin 3 (UCN3) from neighboring endocrine cells [7, 8]. In live animals, pancreatic islets are enriched with different nerve terminals, in which neurotransmitters such as catecholamines and adrenaline profoundly affect insulin secretion [9, 10]. Pancreatic islets also require densely vascularized blood vessels to supply oxygen and nutrients [11], from which pericytes [12] and endothelial cells [13] play crucial roles in islet function. Finally, hormones released from other organs to the plasma, such as glucagon-like peptide 1 (GLP-1) and glucose-dependent insulinotropic polypeptide (GIP), also dynamically impact β-cell secretion via the bloodstream during both normal state and disease progression [14, 15]. Therefore, insulin secretion is precisely regulated across multiple scales, from single cells and pancreatic islets to the intact pancreas and the entire organism.

In addition to IR, reduced immediate responses of insulin secretion upon glucose elevation are another hallmark of T2D [16, 17]. Over the past 50 years, advancements in technology have enabled investigators to explore the underlying mechanisms using a combination of molecular biology, electrophysiology, biochemistry, and mathematical modeling [16, 18]. Although many critical molecules were identified through reductionistic approaches, including experiments with single β-cells and genetically modified animals, the interactions across cell, tissue, and organ levels over time and space that together assemble the physiological glucose-stimulated insulin secretion (GSIS) *in vivo* remain unclear. In this review, we attempt to trace this history and show how high-resolution multi-scale fluorescence imaging techniques help to provide a holistic view of GSIS [19].

## The identification of biphasic GSIS in the pancreas and the mathematical models

In 1960, Yalow and Berson developed an insulin radioimmunoassay (RIA), which enabled accurate measurement and comparison of endogenous plasma insulin from healthy and diabetic human subjects [20]. Using the technique, Curry *et al*. found in 1968 that an abrupt elevation in glucose induces two phases of insulin release in the perfused pancreas [21]. The biphasic GSIS consists of a rapid, but transient, release of insulin (the first phase), followed by a progressively increasing release (the second phase) over 1–2 h. After identifying this phenomenon, Grodsky single-handedly proposed a mathematical model, termed the storage-limited model [22]. According to the model, the first phase of insulin release results from a small but readily releasable component, while the second phase is driven by a larger but slower-releasing component. In addition, these liable components store insulin as packets with a bell-shaped distribution of thresholds to glucose, contributing to the dose-dependent effect of the first phase [22] (Fig. 2). Beyond the general agreement between simulations and experimental results, the model also predicted the disappearance of phasic GSIS once glucose was perfused as slowly increasing ramp functions, mirroring the gradual rise in plasma glucose levels seen during the ingestion of regular meals. Despite these accurate predictions, the GSIS model can not explain the precise nature of these insulin packets.

On the other hand, Nesher and Cerasi demonstrated that successive 40-min stimuli with glucose separated by a 20-min rest period in rat pancreas resulted in more than two-fold potentiation of the first phase of the second response, thus termed time-dependent potentiation (TDP) [23]. In contrast, successive short stimuli with glucose led to an inhibition of the response to the second stimulus, known as time-dependent inhibition (TDI) [23]. Moreover, a linear relationship was observed between the degree of TDP and the slope of the second-phase insulin release, suggesting that both phases of GSIS may have the same origin but are differentially modulated. This finding contrasts sharply with the storage-limited model, which posits that insulin releases during the first and second phases of GSIS have different properties. Based on these observations, Nesher and Cerasi proposed an alternative signal-limited model—the biphasic GSIS is the dynamic interaction between stimulatory and inhibitory signals initiated by glucose, each of them having its own kinetics and dose dependences. While the immediate, first-phase response results from the acute stimulus-secretion coupling, the decay in the first phase of secretion is due to the TDI of insulin release, and the TDP of insulin release is responsible for the slow-rising second phase of GSIS [23].

## Biphasic GSIS is crucial for maintaining glucose homeostasis *in vivo*

In responding to a single-step glucose challenge, biphasic GSIS has been observed in both the islets [24] and the pancreas [22]. A biphasic insulin secretion can only be detected in plasma following a rapid intravenous glucose administration during a hyperglycemic clamp [25]. Because plasma glucose does not rise abruptly during a mixed meal, peripheral insulin concentration does not exhibit a typical biphasic insulin secretion. *In vivo*, insulin concentrations in blood oscillate at a periodicity of 5–15 min [26, 27]. Although the classical first phase of insulin secretion does not occur under physiological conditions, its most likely physiological counterpart is the early phase of insulin secretion, defined as the increase in insulin within the first 30 min after a meal. The first phase of insulin secretion is regarded as the most sensitive indicator of proper β-cell function. It represents the ability to generate a rapidly increasing insulin profile and is particularly effective in restraining hepatic glucose production and limiting postprandial glucose elevation [28]. Thanks to the development of biochemical immunoassays for plasma insulin, it is widely believed that a diminished first-phase insulin release is the earliest detectable defect of β-cell function in individuals destined to develop T2D [16, 17, 29].

For example, in 1968, researchers discovered that the early phase of insulin secretion is impaired in nonobese individuals with a family history of diabetes [30]. Abnormal first-phase insulin secretion is also an early event in the deterioration of β-cell function observed in women with a history of gestational diabetes, even when their postpregnancy glucose tolerance is normal [31]. Recently, new therapy findings indicated that enhancing the first phase through Roux-en-Y gastric bypass surgery can immediately improve glucose tolerance in patients with T2D, despite unchanged glucose effectiveness [32]. This may explain the significant and enduring interest in the biphasic pattern of GSIS in both clinical and basic medical research, from the past till the present.

## Identifying readily releasable pool of vesicles in the stimulation-secretion coupling of β-cells

Despite advancements in understanding the phenotypes, models, and broader significance of β-cell function, exploring the underlying molecular mechanisms remains challenging without isolating single β-cells from islets, optimizing their culture conditions, and evaluating their individual secretory functions. One influential technique, patch-clamp recording, was developed by Erwin Neher and Bert Sakmann in 1976 [33], a breakthrough for which they were awarded the Nobel Prize in Physiology in 1991. The original technique was used to measure the conductance of a single ion channel on the plasma membrane under the cell-attached configuration [33]. As the cell membrane can be modeled as the combination of resistors and capacitors in the electric circuit, the fusion of insulin granules with the plasma membrane increases the cell membrane and causes elevations in membrane capacitance [34]. Thus, fluctuations in cell membrane capacitances could be measured by depolarizing cells with a sinusoidal wave superposing on a square wave of holding potential under the whole-cell configuration (membrane capacitance recording [35]). In fact, individual step increases in membrane capacitance resembling individual granule fusion can be resolved in chromaffin cells [35], mast cells [36], and other endocrine cells. On the other hand, Wightman *et al*. replaced the glass patch pipette with a carbon fiber probe positioned in close proximity to the cell membrane. Upon granule fusion, secreted oxidizable molecules (such as catecholamines, adrenaline, dopamine, and serotonin) can be oxidized by the probe held at a positive voltage, creating a current flow proportional to their local concentrations (amperometry recording [37, 38]). Although both electrophysiological methods can detect quantal exocytosis from single vesicles/granules with similar sensitivity, they reveal different aspects of vesicle fusion [39]. Using these techniques in chromaffin cells, Erwin Neher and colleagues have found phasic and sustained secretion in single chromaffin cells [35, 40], which were later confirmed in other secretory cells, including neurons [41]. Thus, they proposed the concept of a readily releasable pool (RRP) and a reserved pool (RP) of vesicles, which correspond to the fast and slow phases of secretion in chromaffin cells, respectively [35, 40]. Similarly, Rorsman *et al*. also identified voltage depolarization-triggered fast and slow exocytotic events in pancreatic β-cells using the membrane capacitance recording technique [42]. Thus, they proposed that the RRP and RP of vesicles may function as the “insulin packets” that masterly regulate the biphasic GSIS [42].

## Molecular mechanisms of glucose stimulation-insulin secretion coupling and the biphasic GSIS

**Figure 1:**
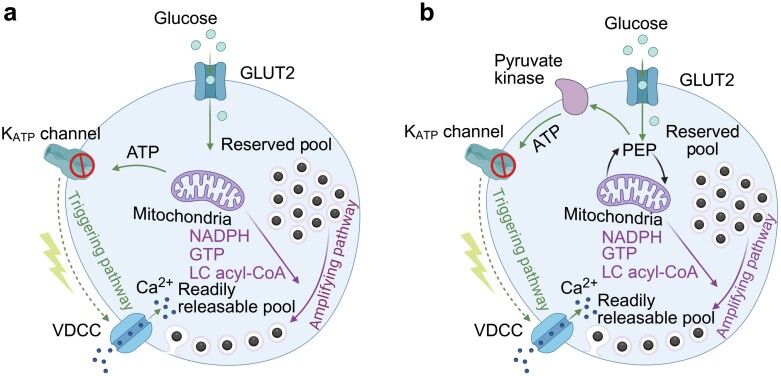
Stimulation-secretion coupling in pancreatic β-cells. (a) Consensus model of GSIS in β-cells. β-cells contain two pools of secretory granules that differ in their release competence. The RRP of granules, available for immediate release, is docked to the plasma membrane and located close to VDCC. The triggering pathway, induced by glucose metabolism, causes Ca²⁺ influx, which evokes the release of granules from the RRP. The RP of granules can refill the RRP through ATP-dependent mobilization and translocation. Mitochondrial metabolites facilitate the replenishment of the RRP from the RP through a process termed the amplifying pathway. (b) A new model of GSIS. In this new model, the closure of K_ATP_ channels is mediated by plasma membrane pyruvate kinase, rather than oxidative phosphorylation in the mitochondria. GLUT2, glucose transporter 2; VDCC, voltage-dependent calcium channel; LC acyl-CoA, long-chain acyl-coenzyme A.

In single β-cells, glucose is transported into the cytosol via glucose-transporter 2 (GLUT2, or GLUT1 in human β-cells [43]), which is crucial for the rapid equilibration of intracellular and extracellular glucose concentrations. After glycolysis facilitated by glucokinase, the metabolites derived from cytosolic glucose undergo further metabolism in mitochondria, generating ATP through oxidative phosphorylation. This increase in ATP leads to the closure of ATP sensitive K^+^ channel (K_ATP_ channel), causing cell depolarization, the opening of voltage-gated calcium channels (VDCCs), and the influx of Ca^2+^, which triggers the release of insulin granules [44] (summarized in Fig. 1a). Recently, the canonical consensus model has been challenged by Merrins’ lab. They propose that the plasma membrane pyruvate kinase, rather than oxidative phosphorylation, acts as the ATP/ADP generator that closes β-cell K_ATP_ channels to initiate insulin secretion [45, 46] (Fig. 1b).

In addition to the electrophysiological characterization of the RRP and RP of vesicles [42], Olofsson *et al*. also used electron microscopy to distinguish the ultrastructural difference between these vesicle pools [47]. About 10% of the granules are docked to the plasma membrane, and up to 30% of the granules are situated within one granule diameter from the plasma membrane [42]. The fast exocytosis reflects the docked granules closed to the membrane [47] and in contact with the L-type Ca^2+^ channels [48]. From the comparison, the estimated size of the RRP is 50–100 granules that can undergo exocytosis per β-cell during first-phase insulin secretion [49]. Using these methods, researchers have begun to identify the molecules required for the biphasic GSIS. For example, consistent with the critical role of L-type Ca^2+^ channels in triggering fast exocytosis, knocking out the Ca^2+^ channel Ca_v_1.2 significantly compromised RRP triggered by depolarization train in individual β-cells. Concurrently, the first phase of GSIS from isolated mouse pancreatic islets was also dramatically diminished [50]. These data, taken together at both the single-cell and islet levels, provide evidence supporting the causal correlations between the RRP and the first phase of GSIS, highlighting Ca_v_1.2 as a key regulator in mouse β-cells. Along similar lines, knocking down Munc18-1, a core protein regulating the soluble *N*-ethylmaleimide-sensitive factor attachment protein receptor (SNARE) complex, also reduced the RRP and the first phase of GSIS [51].

In chromaffin cells, the slow exocytotic component is evoked by depolarization trains to involve the mobilization of RP vesicles and their replenishment into the RRP pool, a process that involves one or several ATP-dependent reactions [35]. Other factors, such as protein kinase C (PKC), have also been associated with this replenishment [52]. Consistent with these experiments, R-type Ca_v_2.3 channels and Munc13-1 have been implicated in the replenishment of RRP in β-cells and are associated with the second phase of GSIS [53, 54]. These molecularly targeted experiments were conducted in β-cells and islets [50, 51, 53, 54], suggesting that the vesicle pools in β-cells correspond well to the storage-limited model. In that sense, the vesicle pool theory predicts the following: (i) the first-phase insulin secretion involves exocytosis of RRP, which constitutes a subset of the docked pool of granules; (ii) RRP comprises a distinct and limited population of granules, and once this pool has been depleted, secretion stops; and (iii) metabolic energy is required for the refilling of RRP from the RP.

On the other hand, as β-cells are uniquely posed to release insulin upon glucose elevation, many researchers have explored their metabolic mechanisms underlying the stimulation-secretion coupling of biphasic GSIS. In the canonical model, it is widely accepted that glucose uses the K_ATP_-dependent pathway to trigger insulin secretion through its direct metabolism [55]. However, Jean-Claude Henquin and his colleagues noted that glucose can increase insulin secretion independently of K_ATP_ channel activation [56]. Therefore, they proposed that there may be two different signaling pathways responsible for insulin release [55, 57]. The triggering pathway involves the closure of K_ATP_ channels and the influx of Ca^2+^, accounting for the first phase of insulin release. Meanwhile, the second phase is K_ATP_ channel-independent but glucose metabolism-dependent, referred to as the amplifying pathway, which augments the efficiency of Ca^2+^ on exocytosis. These two pathways are hierarchical. The triggering pathway predominates over the amplifying pathway, which remains functionally silent as long as Ca^2+^ has not been elevated by the triggering pathway; in other words, as long as glucose has not reached its threshold concentration. The amplifying pathway permits a cytosolic Ca^2+^ rise in β-cells to induce a sustained release of insulin and is a major contributor to the second phase of nutrient-induced insulin secretion. Incorporating the vesicle pool theory, the triggering pathway primarily releases vesicles from the RRP by increasing cytosolic Ca^2+^. In contrast, the amplifying pathway enhances the release of vesicles supplied from the RP, mediated by metabolites derived from glucose metabolism, such as NADPH, acetyl-CoA, and long-chain acyl-CoA (Fig. 1a) [55, 57]. These metabolites also promote the production of rate-limiting factors such as exchange protein directly activated by cAMP (EPAC), protein kinase A (PKA), PKC, and diacylglycerol (DAG), thereby enhancing the vesicle fusion machinery [58, 59].

Additionally, Sharp and his colleagues posited that the nadir between two phases of insulin secretion may be controlled by certain rate-limiting steps [60, 61]. They theorized that docked granules vary in their ability to be released, forming two subsets: the RRP and the immediately releasable pool (IRP). Granules from the IRP are the first to be secreted in response to an increase in intracellular Ca^2+^ during the triggering pathway, resulting in the first phase of insulin secretion. At the nadir of secretion between the two phases, granules from the RRP are converted to those in the IRP through an ATP-dependent process known as “priming”. This priming step is suggested to be the rate-limiting step for exocytosis and the target for signals in the amplifying pathway, which leads to the sustained second phase of insulin secretion. By the efforts of an enormous number of researchers, currently the consensus model is that the triggering pathway evokes the vesicles in RRP contributing to the first phase, while the amplifying pathway maintains the release of the second phase by mobilization of vesicles from RP to RRP [62] (Fig. 1a).

## Fluorescence imaging reveals the heterogeneity in insulin granule exocytosis

Despite the prevailing hypothesis that the RRP underpins the first phase of GSIS, an issue arises from the observation that different glucose concentrations trigger different peaks of the first phase of GSIS [22]. This variability casts doubt on the notion, given that RRP vesicles, due to their proximity to the plasma membrane, would presumably be of limited and consistent size [49], and not significantly altered by acute glucose application. To address this issue, advanced imaging techniques are required to visualize these RRP vesicles directly within live cells.

One such technique is modern total internal reflection fluorescence microscopy (TIRFM), which enables the observation of a specimen’s region thinner than 100 nm. TIRFM was developed by Daniel Axelrod in 1980 [63]. However, it was not until the development of commercial oil objectives with a numerical aperture (NA) higher than 1.4 that TIRFM became a standard technology for cell biological studies. Equipped with a 1.65 NA oil objective, Steyer *et al*. have used TIRFM to directly visualize the motility and exocytosis of individual granules beneath the plasma membrane in chromaffin cells. In these cells, the RRP measured electrophysiologically corresponds to a small subset of docked granules observable with TIRFM [64]. Similarly, Shibasaki *et al*. analyzed insulin granule dynamics in primary mouse pancreatic β-cells [58]. They identified three types of insulin-fused granules: the “old face,” where predocked granules fuse immediately with the plasma membrane upon stimulation; the “restless newcomer,” where newly recruited granules are immediately fused to the plasma membrane upon stimulation; and the “resting newcomer”, where newly recruited granules are first docked before subsequent fusion. In their opinion, the vesicle pool hypothesis should be revised, as both the first and second phases of GSIS are dominated by the restless newcomer [58]. On the other hand, granule aging is another important factor in the selection of insulin granules for secretion. Evidence dating back to pulse-chase radiolabeling studies in the 1960s [65] suggests that newly synthesized insulin granules are preferentially released during glucose stimulation [66, 67]. This challenges the understanding that the “old face” granules, which are pre-docked insulin granules near the cell membrane, predominantly contribute to the first phase due to their readiness for immediate release [68].

Alternatively, Tsuboi *et al*. have also used TIRFM to reveal various insulin granule fusion modes, such as “kiss and run” [69] and “cavicapture” [70], with the noncomplete fusion mode further confirmed by the amperometric method [71]. While “kiss and run” may regulate the release of small transmitters [72], how much these incomplete vesicle fusion events contribute to the overall insulin secretion remains unclear. Hence, the relationship between RRP and RP defined by the electrophysiological methods with biphasic GSIS measured in islets and pancreas may not be as straightforward as previously thought.

## Omics techniques reveal molecular and functional heterogeneity in β-cells

Since 1986, it has been understood that β-cells are heterogeneous in regards to insulin synthesis [73], glucose responsiveness (cytosolic Ca^2+^ concentrations [74] and NADPH [75]), and insulin secretion [76, 77]. This heterogeneity has become even more evident with the development and application of various single-cell omics techniques over the past decade. Leveraging these high-throughput single-cell omics methods, several landmark studies have uncovered significant variations in gene expression among individual β-cells isolated from rodent or human islets [78–82]. Numerous subpopulations of β-cells, differentiated by molecular markers or functional properties, have been identified, and their proportions vary between healthy individuals and patients with T2D or other islet dysfunction conditions (review in reference [83–85]). For example, Rubio-Navarro *et al*. have used single-cell RNA sequencing (scRNA-seq) to identify a subset of β-cells enriched with CD63, which show higher expression of mitochondrial metabolism genes and more pronounced GSIS [78]. Alternatively, the use of additional surface markers like CD9/ST8SIA1 for sorting β-cells has led to the identification of a subpopulation with enhanced insulin secretion capability [82]. Consistent with this variation in secretory ability, three β-cell subpopulations with distinct redox statuses have been identified [86]. Finally, research has shown that epigenetic dosage acts as a novel regulator of cell subtype specification, revealing two functionally distinct β-cell subtypes [79].

Despite the heterogeneity revealed by omics methods, the functional roles of these subpopulations within intact islets, especially *in vivo*, remain unclear. To address this, new techniques are needed that can assess the function of single β-cells within islets or *in vivo*. Indeed, large-scale fluorescence Ca^2+^ imaging technique that uses Ca^2+^ signals as a surrogate of β-cell function has revealed the presence of functional β-cells within live islets. By combining Ca^2+^ imaging with network analysis, researchers have found specific cells within islets known as hubs [87], leader cells [88], or first responder cells [89] in islets, which appear to modulate responses of other neighboring β-cells to glucose stimulation [84]. For example, 1%–10% of β-cells in mouse islets that showed this “hub” behavior revealed important properties related to islet function [87]. These hub cells showed immature signatures (low pancreatic duodenum homeobox-1 (PDX1) and insulin) but increased glucokinase levels, thereby indicating enhanced metabolic activity. Notably, strong optogenetic-mediated hyperpolarization of these hub cells in mouse islets resulted in suppression of both islet glucose-stimulated [Ca^2+^] levels and insulin release [87]. Modulating the ratio of immature to mature cells also altered islet function [90]. Similarly, photoablation of zebrafish leader cells disrupted pan-islet signaling [88], while ablation of the first responder cells slowed down, diminished, and discoordinated the first phase of [Ca^2+^] transients [89]. Consequently, it is expected that certain β-cells may have a more significant impact on overall insulin release than others. Furthermore, Ca^2+^ imaging of mouse pancreas tissue slices confirmed that islets exhibit small-world properties of functional connectivity, especially under stimulation [91, 92]. Additionally, we and Ninov’s lab have also shown that during the development of zebrafish pancreas, β-cells have different functional properties. For example, immature β-cells in the islet core and mantle of the living zebrafish embryo display different glucose-stimulated Ca^2+^ signals [93]. The older and younger β-cells occupy different regions within the islet, with the former exhibiting robust glucose responsiveness whereas the latter being more proliferative but less functional [94]. Therefore, it remains obscure how heterogenous Ca^2+^ signals lead to diverse functions in insulin exocytosis. The extent of control that β-cell subpopulations exert over overall islet function still needs to be thoroughly explored.

Interestingly, the conventional biphasic GSIS mechanism, attributed to the RRP in β-cells, assumes that all β-cells within the islet function similarly and their exocytotic events are equally effective in releasing insulin. Under such conditions, the biphasic GSIS in islets or pancreas can be approximated as the spatiotemporal sum of all fusion events from individual β-cells. However, given the significant heterogeneity in insulin exocytosis and β-cell function within islets, it is necessary to reexamine the basis of GSIS, which requires new methods to integrate dynamic information from individual vesicular fusion events up to the islet level.

## A new mechanism of biphasic GSIS: zinc sparkle imaging enables a trans-scale, integrated imaging across islet

To study GSIS, a high-resolution imaging method to visualize individual vesicle exocytosis is essential. Researchers have combined two-photon imaging with the use of noncell-permeable fluorescent probes, which could be used to visualize individual secretion events within islets [95, 96]. Using this approach, fusion pore dynamics [95] and insulin release sites towards the vasculature [96] have been revealed. However, it often captures only a limited number of fusion events [95, 96]. On the other hand, insulin granules are enriched with zinc, which is co-released with insulin during exocytosis and can be visualized using a nonpermeable fluorescent zinc indicator in isolated single β-cells [97]. Although Li *et al*. have developed a fluorescent, cell surface-targeted zinc indicator for monitoring induced exocytotic release (ZIMIR), which was used to monitor secretion within the islet, such a method lacks the spatiotemporal resolution to simultaneously visualize individual fusion events and provide a comprehensive summary [98]. In addition to using zinc probes, granule-targeted, pH-sensitive probes such as neuropeptide Y (NPY)-pHluorin could be used to label insulin granules, while disc-spinning confocal microscopy has been used to monitor secretion events within the islet [99]. The problem again, however, was the low detection rate of fusion events by such a method, preventing the method from quantitatively exploring the biphasic GSIS in islets.

By combining the cell-impermeable zinc dyes (FluoZin-1, PKZnR-1, and PKZnR-5) with high-speed, high-resolution disc-spinning confocal microscopy, we have recently developed a method that visualizes hundreds of insulin granule fusion events (“sparkles”) from β-cells within a single intact islet. Additionally, we developed an algorithmic pipeline to unbiasedly detect and categorize these numerous fusion events. As a result, we obtained a spatiotemporal ensemble secretion profile, which exhibited a rapid peak followed by multiple smaller peaks, closely resembling the biphasic GSIS measured by enzyme-linked immunosorbent assay (ELISA). Most importantly, we demonstrated that different glucose concentrations triggered the first peak with varying amplitudes, while the secretion induced by 29.2 mmol/L glucose approximated that produced by the sequential application of 9.8, 18.2, and 29.2 mmol/L glucose [19]. This data is consistent with GSIS measurements by RIA in the perfused pancreas [21], underscoring that our high-resolution imaging method detected insulin secretion in a manner quantitatively comparable to the gold standard of ELISA and RIA for the first time. This capability is essential for the next stage of dissecting biphasic GSIS at the cellular and vesicular levels.

**Figure 2:**
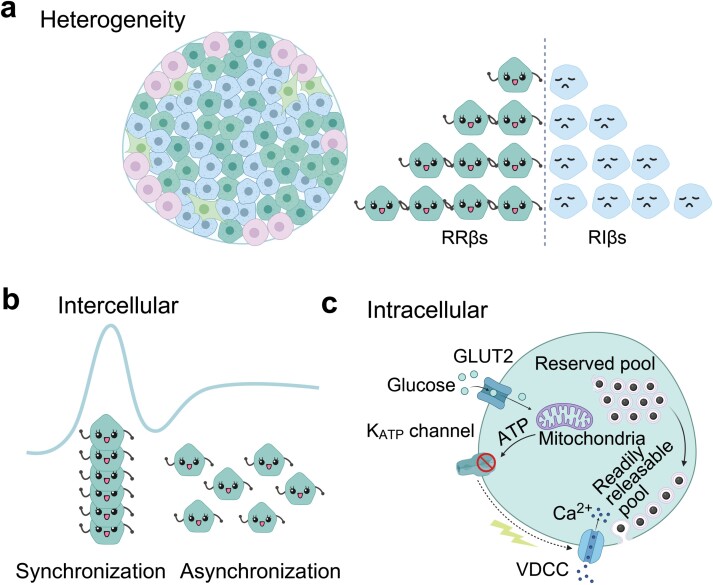
The intercellular and intracellular processes of biphasic GSIS. (a) β-cells within intact islets can be categorized into RRβs and RIβs. (b) The synchronization and asynchronization of RRβs determines different phases of GSIS over islet. (c) Within a single β-cell, RRP granules in RRβs account for the first phase, while the replenishment of RRP from RP granules contributes to the second phase.

Next, glucose stimulation triggered significant heterogeneous secretory responses from individual β-cells within the islet, following an exponential distribution. This suggested that a minority of β-cells contributed to the majority of the secretion, a finding further confirmed by evaluation using the Gini coefficient. Therefore, we defined the highly secretory β-cells that contributed to 80% of the total insulin secretion as readily releasable β-cells (RRβs), and others as releasable-incompetent β-cells (RIβs). Surprisingly, we found that it is the RRβs, not the RRP vesicles, that regulate both phases of GSIS. Specially, biphasic GSIS appears to be regulated at the islet level by three mechanisms (summarized in Fig. 2): (i) The RRβs subpopulation dominates different phases; (ii) The first phase is mainly determined by the synchronization of RRβs, while the second phase results from their asynchronization; and (iii) The synchronized release of RRP granules in RRβs accounts for the first phase, while the replenishment of RRP from RP granules may contribute to the second phase, especially at supraphysiological doses of glucose stimulation.

## Another lesson learned: Ca^2+^ signals may not always be the faithful surrogate for islet β-cell function in health and disease

Notably, we did not observe significant differences in glucose-stimulated Ca^2+^ signals between RRβs and RIβs, despite a more than 7-fold difference in exocytosis [19]. This argues against the presumed role of Ca_v_1.2 and Ca_v_2.3 in mouse [50, 53] or Ca_v_2.1 in human islets [100] in mediating biphasic GSIS. Moreover, we found that approximately 60% of the “first-responder cells” [89] or “leader cells” [88] were RRβs, while only about 30% of the “second-follower cells or last responder cells” [89] or “hub cells” [87] were RRβs. Taken together, these data suggest that the exponential distribution of insulin secretory capability is not driven by the heterogeneous connectivity of Ca^2+^ signals in the islet β-cells. Alternatively, these heterogeneous Ca^2+^ signals may differently affect β-cell intrinsic properties, such as endoplasmic reticulum (ER) stress [101], maturation [102], and mitochondrial metabolism [90]. Nevertheless, the decoupling of glucose-stimulated Ca^2+^ signals from insulin secretion is puzzling and warrants future exploration.

Interestingly, compared to control islets, *ob/ob* islets exhibited a significantly reduced proportion of RRβs, making up approximately 26% of islet cells in four-week-old mice and around 27% in adult mice [19]. In adolescent mice, these RRβs demonstrated increased insulin secretion to compensate for their reduced numbers; however, their secretory capacity progressively decreased with age. The *ob/ob* islets demonstrated low amplitude but spiked Ca^2+^ dynamics compared to the control islets, which more efficiently triggered bursts of insulin secretion, particularly during the second phase [19]. Therefore, this enhanced stimulation-exocytosis coupling in young *ob/ob* islets may originate from the spike form but not the absolute amplitudes of Ca^2+^ transients *per se*.

**Figure 3:**
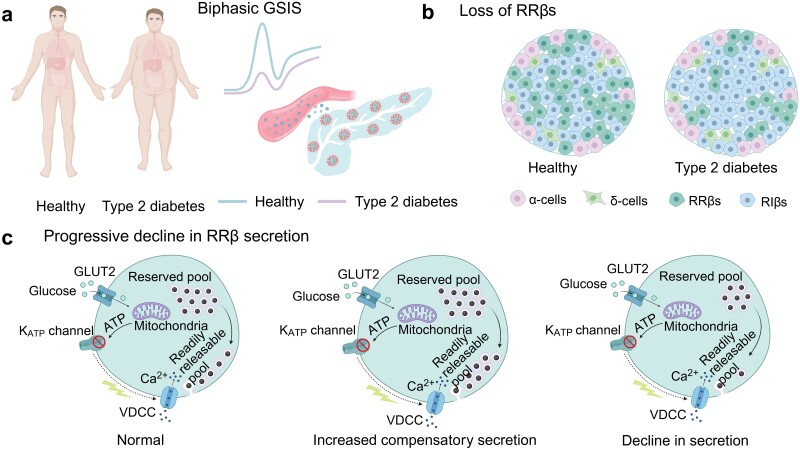
The mechanism underlying impaired biphasic GSIS. (a) Loss of the first phase is a hallmark of T2D patients. (b) In intact islets, reduced RRβs dictate the diminution of the first phase. (c) Progressive declines in the enhanced secretory capability of individual RRβs contribute to the insufficient compensation of GSIS.

Therefore, we demonstrate that the heterogeneous secretory capability of islet β-cells plays a central role in the progression of T2D. The initial impaired first phase may be attributed to the loss of RRβs (possibly due to the dysfunction of paracrine regulation discussed later), whereas declines in the enhanced secretory capability of individual RRβs contribute to the insufficient compensation of GSIS (Fig. 3). Furthermore, we argue that Ca^2+^ signals may not always be a reliable surrogate for β-cell function, and extra caution should be taken to confirm its correlation with insulin secretion. Overall, this holistic and systematic view of the biphasic mechanism and disease pathology may help identify the true causes of this complex metabolic disease.

## The unresolved issue regarding the β-cell functional heterogeneity: complex paracrine interactions within the islet may be the key

It remains unclear why β-cells demonstrate an exponential distribution of secretory capability in the intact islet. Beyond intrinsic heterogeneity, insulin secretion may also be significantly modulated by hormones and neurotransmitters released from neighboring endocrine cells in the islet. Functionally, mouse α-cells secrete glucagon to potentiate insulin secretion [103], while human α-cells also secrete acetylcholine to prime β-cells and potentiate GSIS [104]. Glucagon released from α-cells, initially believed to solely mediate glycogen breakdown in the liver, has also been found to enhance insulin secretion via both GLP-1 receptor and glucagon receptor pathways [103, 105–107]. This paracrine effect is physiologically relevant, as the relative pathway potency shifts from the GLP-1 receptor pathway in normal mice to the glucagon receptor pathway in high-fat diet-fed mice [103]. On the other hand, pancreatic β- and δ-cells are usually regarded as inhibitory factors by releasing insulin and SST to inhibit surrounding cells [7, 8, 108]. However, β-cells may also release UCN3 to activate δ-cells, thus temporally limiting the manifestation of biphasic GSIS [109] (predictably, the impaired SST secretion enables exaggerated first- and second-phase GSIS [110], which is also immediately normalized upon perfusion with synthetic UCN3). Alternatively, δ-cells may connect to β-cells via gap junction and promote activities of some islet β-cells at glucose concentrations close to the rest condition [111].

Thus, we tested the hypothesis that δ-cells may clamp the neighboring β-cells in the islet. Applying the SST type 2 receptor (SSTR2) antagonist CYN-154806 or exogenous SST increased or decreased the number of RRβs and the first phase of GSIS, respectively. This suggests that δ-cells may prevent some β-cells that display normal glucose-stimulated Ca^2+^ signals from triggering insulin secretion. However, the lack of consistent enhancement in insulin secretion upon adding MK-4256, an SSTR3 antagonist, complicates this hypothesis. SSTR3 is selectively expressed in mouse β-cells, while SSTR2 is selectively expressed in mouse α-cells at the transcript level [112, 113], suggesting that pancreatic α-cells may also play a role in establishing β-cell secretory heterogeneity within the islet. Additionally, CYN-154806 failed to stimulate insulin secretion in islets that have a high number of δ-cells. Islet architecture itself impacts how cells interact and communicate, thereby influencing overall islet function. For instance, in rodent islets, extensive interactions primarily occur between β-cells at the core, while interactions between these β-cells and the α-cells and δ-cells are more frequent on the periphery [2]. However, this structural organization varies between species. There is a more complex and diverse architecture in human islets, characterized by increased numbers of heterotypic contacts between β-cells and non-β-cells and fewer homotypic contacts between β-cells [2, 114]. Therefore, the nonoverlapping β-cell functional heterogeneity in terms of secretion and Ca^2+^ signaling within the islet is likely due to the combined, differential effects of the quantity, functional interplay, and spatial arrangements of the α- and δ-cells within the islet.

## Summary of advantages and disadvantages of the methods mentioned

The study of pancreatic β-cell physiology and pathophysiology is always impelled by the emergence of new technologies. The application of single-cell approaches in evaluating β-cell Ca^2+^ signaling and exocytosis, particularly electrophysiology, has led to many important discoveries. For example, identifying K_ATP_ channels through patch-clamp electrophysiology [115] led to the discovery of *KCNJ11* (the gene encoding the Kir6.2 subunit of the β-cell K_ATP_ channel) mutations associated with neonatal diabetes in humans [116]. This discovery was followed by a successful transition from insulin to sulfonylurea treatment, showcasing a precise therapy approach [117]. However, such methods are sometimes incapable of precisely depicting β-cell function and heterogeneity within their native environment: the assembly of multiple endocrine cells into the islet. The emergence of high-resolution imaging methods aims to resolve this problem by providing a detailed mapping of insulin secretion within intact islets. The combination of two-photon microscopy with the use of non-cell-permeable fluorescent probes has shown a good correlation between the number of granule fusion events detected and the measured amount of insulin secretion. It has also demonstrated some extent of partial recruitment of islet β-cells by glucose [118]. However, the limited number of fusion events detected may prevent the acquisition of a complete and quantitative biphasic GSIS profile. This is where the newly developed Zn^2+^ imaging method excels, in addition to its specificity in detecting the release of insulin/Zn^2+^ crystals from insulin granules [19]. However, it may not detect secretion from insulin granules devoid of zinc, such as those found in the β-cells of guinea pigs [119]. Additionally, it lacks the ability to observe granule dynamics associated with priming and docking. The hybrid tagging of endogenous insulin with SNAP/HALO-tag, as developed by Solimena lab [120], may address these issues, though it comes at the cost of creating transgenic animals or using viral infections. In this respect, the Zn^2+^ imaging method, which bypasses the need for prior genetic labeling, is more readily applicable for evaluating islet functions from diverse sources, including human patients. With its capability to link insulin granule releases with individual β-cell functioning in intact islets, we believe that many longstanding questions could be reinvestigated from a new perspective.

## Future perspectives

From these new discoveries, we have come to understand that the overall function of an islet should be considered as the integrated sum of all intracellular molecules and organelles, as well as intercellular communications and coordination. Along these lines, we conclude that biphasic GSIS is an emergent property of the islet level or higher, which cannot be adequately explored at the cellular level alone. Notably, our current hypothesis that RRβs dictate biphasic insulin secretion is primarily derived from studies on isolated islets stimulated with glucose. Recently, it has been found that nutrients such as amino acids and fatty acids also affect biphasic GSIS both *in vivo* and *in vitro* [7, 121, 122]. How these nutrients, beyond glucose, directly regulate RRβs and RIβs or indirectly modulate α-cells and δ-cells to impact biphasic GSIS, remains to be defined.

Furthermore, isolated islets lack blood vessel vascularization and nerve innervation. In mouse islets, parasympathetic and sympathetic axons innervate α-, β-, and δ-cells. In contrast, human islets exhibit minimal endocrine cell innervation, with sympathetic axons preferentially contacting smooth muscle cells of the vasculature [10]. Thus, the autonomic nervous system may regulate human islet function via sympathetic input acting on contractile cells within the vasculature of the islet, altering local blood flow, or by using the vascular route to influence endocrine cells located downstream from the release sites [123–125]. In agreement with these species-specific differences in islet ultrastructure, the second phase of GSIS varies between rats, humans, and mice. Therefore, understanding how these intercellular and inter-tissular communications affect biphasic GSIS across different species *in vivo* remains a challenging yet crucial area for future exploration. To truly understand the causal emergence at various scales, we argue that imaging methods encompassing multiple scales while retaining high spatiotemporal resolution, along with unbiased image data analysis algorithms, are required.

## Conclusions

Since the first report of biphasic insulin secretion by Curry *et al*. [21], the field has experienced evolving research directions over the past 50 years. These have included studies measuring insulin *in situ* from the pancreas and *in vivo*, to the detection of single vesicle exocytosis in isolated single cells, and then back to *in situ* high-resolution imaging of islet functions. While many critical elements have been identified for insulin secretion, the collective modulation of this process by other islet cells and organs—such as the blood vessels, the endocrine system, and even the brain—suggests that these components play a crucial role at a physiologically relevant level. In an era when novel techniques in single-cell and spatial transcriptomics, proteomics, and metabolomics are emerging daily, we emphasize the importance of contextualizing these molecules within their physiological space and time. Achieving this will require new cross-scale imaging techniques and advanced data analysis capabilities. By combining these approaches, we aim to gain a holistic and systemic understanding of how biphasic GSIS occurs and its physiological role in glucose homeostasis.
